# A predictive account of how novelty influences declarative memory

**DOI:** 10.1016/j.nlm.2021.107382

**Published:** 2021-03

**Authors:** Jörn Alexander Quent, Richard N. Henson, Andrea Greve

**Affiliations:** a MRC Cognition and Brain Sciences Unit, University of Cambridge, United Kingdom; b Department of Psychiatry, University of Cambridge, United Kingdom

**Keywords:** Novelty, Memory, Predictive coding, Prediction error, Surprise, PIMMS

## Abstract

A rich body of studies in the human and non-human literature has examined the question how novelty influences memory. For a variety of different stimuli, ranging from simple objects and words to vastly complex scenarios, the literature reports that novelty improves memory in some cases, but impairs memory in other cases. In recent attempts to reconcile these conflicting findings, novelty has been divided into different subtypes, such as relative versus absolute novelty, or stimulus versus contextual novelty. Nevertheless, a single overarching theory of novelty and memory has been difficult to attain, probably due to the complexities in the interactions among stimuli, environmental factors (e.g., spatial and temporal context) and level of prior knowledge (but see Duszkiewicz et al., 2019, Kafkas and Montaldi, 2018b, Schomaker and Meeter, 2015). Here we describe how a predictive coding framework might be able to shed new light on different types of novelty and how they affect declarative memory in humans. More precisely, we consider how prior expectations modulate the influence of novelty on encoding episodes into memory, e.g., in terms of surprise, and how novelty/surprise affect memory for surrounding information. By reviewing a range of behavioural findings and their possible underlying neurobiological mechanisms, we highlight where a predictive coding framework succeeds and where it appears to struggle.

## Highlights

• A single overarching theory of novelty and memory has been difficult to attain.
• Conflicting findings showing that novelty can enhance and impair memory in cases.
• Predictive coding framework is able to shed new light on different types of novelty.
• This has important consequences for the effect of novelty on declarative memory.

## 1 Introduction

Events that are new, different or unusual often “stick in your mind”. For example, imagine you live and work in an urban area and you take the same route to work in your car every morning. One day you see a flock of sheep blocking the road on your commute. You will probably remember this event for a long time, while the other countless times you have driven down that road are forgotten. Numerous studies have confirmed this observation, namely that if we experience a novel event in a familiar context, we tend to store and remember this event more easily. However, if you happen to live in the countryside close to a sheep farm, your experience might be quite different: because you see flocks of sheep quite frequently, you might experience just another regular commute to work that is hardly memorable. Thus, the experience of novelty is not ‘absolute’ and cannot be defined independent of the observer; rather it is driven by what an individual expects to experience compared to what they actually encounter. This comparison between expected and experienced outcomes parallels the computation of a prediction error (PE) in many theories of learning. According to such theories, we continuously generate expectations about our environment, and update those predictions when they are wrong, i.e., when a PE occurs. While this role of PE in learning is well established in experiments on non-declarative memory (e.g., in conditioning and associative learning in animals), its role in human declarative memory (i.e., conscious, verbalisable memory; Squire, 2004) is less well established. In line with a recent review by Reichardt et al. (2020), we propose that predictive coding theories in general, and the ‘Predictive Interactive Multiple Memory Signals’ (PIMMS) proposal of Henson and Gagnepain (2010) in particular, provide a powerful framework to help us understand how novelty influences declarative memory. Going beyond Reichardt et al. (2020), we show how PIMMS reveals multiple different types of novelty, including situations that lead to poor memory, and how it allows for PE at different levels of representation, which might map onto different types of memory (e.g., recollection versus familiarity) that involve distinct brain regions. In doing so, we also reveal empirical findings that are not addressed by PIMMS, such as the effect of novelty on surrounding information, and a memory advantage for expected information (with little apparent PE).

## 2 Novelty and prediction errors

The question of how we remember novel information has inspired numerous studies over the past decades. Berlyne (1960) was the first to introduce the term *absolute novelty* for something that has never been encountered before, as distinct from *relative novelty,* which describes novel arrangements of familiar elements. More recent accounts use the term *stimulus novelty* to refer to absolute novelty, and contrast this with *contextual novelty* (Ranganath and Rainer, 2003, Schomaker and Meeter, 2015), which arises when a familiar item is encountered in an unexpected context (more akin to Berlyne’s relative novelty). Regardless of the terminology, what these concepts have in common is that both arise against a backdrop of (the presence or absence of) prior expectations. The PIMMS framework offers one way to think about the relationship between the precision of predictions and the uncertainty of sensory input.

According to PIMMS, memory and perception arise within multiple levels of a processing hierarchy, where higher levels are constantly predicting the activity in lower levels, and the difference between the predicted and actual activity (at a single moment in time) – the PE – is fed back from lower to higher levels. For a given layer, the predictions from the level above are equivalent to a “prior” probability distribution (in a Bayesian sense), whereas the activity profile (produced from the level below) is equivalent to the evidence or “likelihood”, while the PE is the *divergence* between these two distributions (the summed area of no overlap)^1^. The size of the PE then determines how well a new event is encoded into memory.

**Fig. 1:**
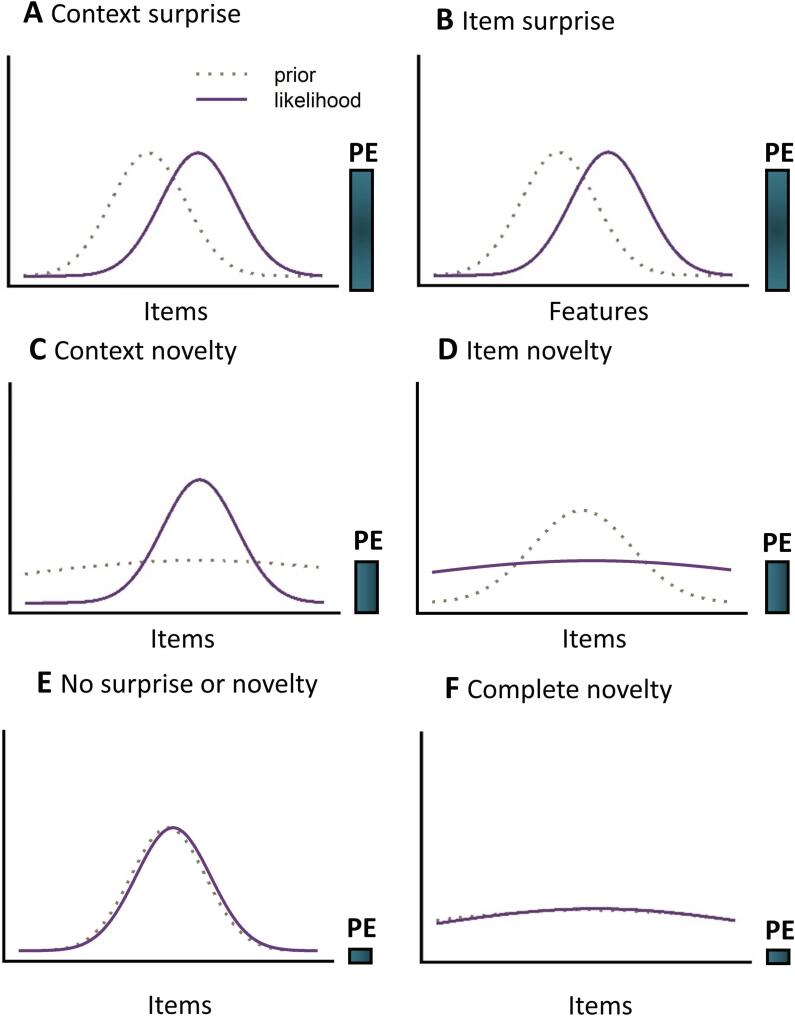
Schematic illustration of prior and likelihood distributions in the “predictive interactive multiple memory signals” framework (PIMMS). Distributions reflect activity in a layer of topographically organised neurons, where the x-axis captures similarity between items in the semantic level or similarity between features in the perceptual layer (depending on the panel). The dotted line represents the prior predictions from the “higher” level, whereas the solid line represents the likelihood distribution, input from the level below (ultimately the sensory input). The PE, which drives learning, is the divergence between these two probability distributions, whose magnitude is illustrated at the bottom right of each panel. Panel A: precise prior and precise likelihood for items in a certain context, but with different modes (context surprise). Panel B: the same as Panel A, except the precise prior and likelihood refer to features of an object in lower perceptual levels (item surprise). Panel C: Flat prior and precise likelihood (context novelty). Panel D: precise prior and flat likelihood (item novelty). Panel E: precise prior and precise likelihood with the same mean (leading to no PE or learning; no surprise or novelty). Panel F: flat prior and flat likelihood, a combination of context novelty and item novelty (or “complete novelty”), but one predicted to show no PE or learning.

Let us consider a level representing the current items perceived (e.g., objects), which may or may not be predicted from a higher level representing the context (e.g., environment). Take the example of sheep encountered in an urban environment: the context is predicting houses, road-signs, other cars, etc., whereas the sensory input is indicating sheep. This corresponds to a prior and likelihood that are quite precise but differ in their modes (Fig. 1A). This results in high PE, which causes strong encoding of the event. We refer to such situations as “surprise”, in keeping with other related work (Reichardt et al., 2020). In fact, this surprise could occur at multiple levels of the processing hierarchy. When it occurs between contextual predictions about familiar items, we will call it “context surprise”. However, our knowledge of familiar items (e.g., sheep) allows us to make predictions about the perceptual features (e.g. four legs, white wool, etc.) that comprise those items. When one or more features differ from those expected (e.g., a pink sheep), then PE occurs at this lower level of the hierarchy. We call this “item surprise” (Fig. 1B); similar to Berlyne’s concept of relative novelty.^2^

Now consider another situation, where you enter a new environment (a context that you have not experienced before), then you have minimal predictions about what items to encounter, such that the prior is imprecise, or in the extreme case, “flat”. We will call this “context novelty”. When you do encounter a familiar object (such that the likelihood is precise), there is still a PE (though not as great as in Fig. 1A/B), which can lead to reasonable memory encoding (Fig. 1C). For example, imagine you had always lived on a remote sheep farm, and entered a city for the first time, where you do not quite know what to expect. If you encountered a flock of sheep, this might be somewhat surprising, even if not as surprising as for the urban commuter considered above.

Or consider a situation where you encounter an item you have never seen before. In this case, the likelihood is flat instead – i.e., you do not know how to interpret the sensory evidence. We call this “item novelty”. When you encounter such an unknown item in a familiar environment, where you are expecting other objects instead (i.e. a precise prior, Fig. 1D), then there is again a small PE. This would correspond to the urban commuter encountering the sheep, but in this case, having no prior knowledge of animals like sheep at all.

Finally, in the case when the prior and the likelihood overlap closely (Fig. 1E), so that PE is low, there is no need to do any learning (i.e., no need to waste resources re-encoding what is already known). However, a low PE can also emerge from a flat prior and flat likelihood, as in Fig. 1F. Importantly, this situation of “complete (or maximal) novelty” (e.g., encountering unknown objects in an unknown environment) is actually predicted to produce negligible, rather than “maximal”, learning.

PIMMS originally distinguished between at least three levels that differ in their representational content: the “episodic” system, at the top of the hierarchy, which stores associations between spatiotemporal contexts and items (objects); a “semantic” level, which contains knowledge about individual items and their corresponding configuration of perceptual features; while lower in the hierarchy, “perceptual” systems represent individual, modality-specific features of the stimuli.

The key function of the episodic system is to optimize the predictability of an item occurring in a particular spatiotemporal context, i.e., store context-item associations. When we encounter a familiar item in a context that is different from that expected, the ensuring PE induces learning of more accurate associations between episodic and semantic representations. This is the type of learning that enables memory of the spatiotemporal context in which an item occurred, or what has been called “recollection” (Mandler, 1980, Montaldi and Mayes, 2010, Yonelinas, 2002). This episodic level is associated with the hippocampus and other brain structures linked with episodic memory (Aggleton and Brown, 1999, Mayes et al., 2007, Moscovitch, 1995, Moscovitch et al., 2016, Scoville and Milner, 1957, Squire, 1992). Note that PEs can, and normally do, arise automatically, based on prior knowledge triggered by perceptual inputs, i.e., predictions are rarely intentional or effortful. However, whereas Morris (2006) claimed that attended experiences are automatically recorded by the hippocampus, but will normally fade and be lost, we claim that PE modulates the degree of encoding and therefore determines whether an experience will be available as a lasting memory.

The semantic level on the other hand stores information about familiar items, and predicts which features are expected on the basis of a given item being present. Note that there is a bidirectional flow of information between all systems, so not only do currently active item representations make predictions about associated perceptual features, but currently active features also influence which item representations remain active (i.e., perception is a dynamic competition across all systems in order to minimise overall PE). The semantic system is associated with anterior temporal lobe regions, including perirhinal cortex, and it is the strengthening of item-feature associations that enables the feeling of “familiarity” (rather than recollection), which sometimes accompanies recognition memory (Mandler, 1980, Montaldi and Mayes, 2010, Yonelinas, 2002).

There are probably numerous levels of intermediate perceptual representations, depending on the modality, but for simplicity we consider posterior temporo-occipital regions associated with perception of visual stimuli. However, since we focus here on declarative memory, we will concentrate on the episodic and semantic systems.

Given this brief overview of PIMMS, we summarise different types of novelty/surprise in Table 1. With these definitions in mind, we will first see how well they can capture the human behavioural literature. Then we will consider how they might relate to neurobiological mechanisms, such as those studied in the animal literature.

**Table 1:** Glossary with the main forms of surprise and novelty.

Term	Definition	PIMMS	Example
Context surprise	When familiar items occur in an unexpected context. Has previously been called “contextual novelty” (Ranganath and Rainer, 2003, Schomaker and Meeter, 2015); though used differently here (see Context Novelty below)	A strong prior from the context level to the item level is accompanied by strong but divergent evidence from item level (Fig. 1A)	An urban commuter encounters a flock of sheep in the city.
Item surprise	When a familiar object that has one or more unexpected features. Analogous to what Berlyne (1960) called relative novelty.	A strong prior from the item level to the feature level is accompanied by strong but divergent evidence for one or more features (Fig. 1B)	A sheep farmer encounters a pink sheep.
Context novelty	The context is so unfamiliar that you do not know what to expect	A flat prior from the context level to item level, i.e., few predictions about the kind of items present. Note that whether or not PE occurs depends on whether the likelihood is precise or also flat (cf. Fig. 1C vs 1F)	A sheep farmer who has never visited a city before.
Item novelty	Items that have not been encountered before. Analogous to what Berlyne (1960) called absolute novelty, or more recent definitions of “stimulus novelty” (Ranganath and Rainer, 2003, Schomaker and Meeter, 2015).	Flat evidence at the item level, i.e., the features present do not activate a unique item representation. Note that whether or not PE occurs depends on whether the prior is precise or also flat (cf. Fig. 1D vs 1F).	An urban commuter encounters a sheep having never seen one before.
Complete novelty	Unknown items encountered in an unknown context. This is a combination of context novelty and item novelty.	Flat prior and flat likelihood (Fig. 1F)	An urban commuter encounters a sheep on a farm, having never been to a farm or seen a sheep before.

## 3 Context surprise

Context surprise is the most studied form of novelty in the human psychological literature. Going back to our initial example, the context we are in (being on the way to work) predicts the presence of certain objects and events. If we now encounter something that is not predicted, like the sheep, then the resulting difference between prediction (the prior) and observation (the likelihood), give rise to a PE, which triggers the formation of an episodic memory for this unexpected event.

### 3.1 Expectations arising from shared episodic context

**Fig. 2:**
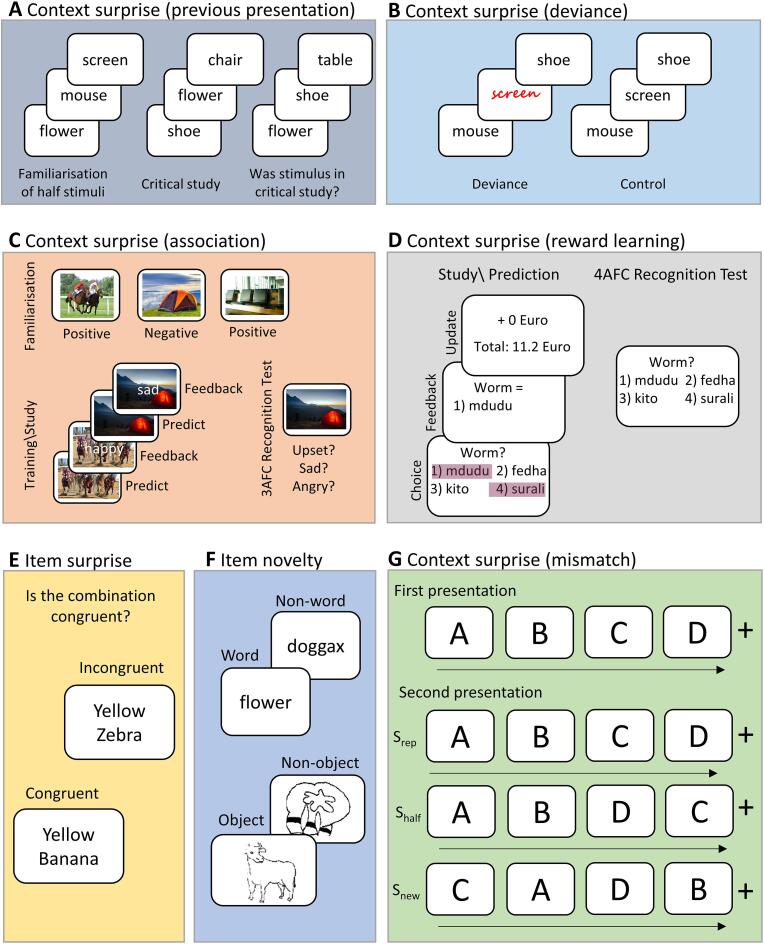
Illustration of key paradigms. Panel A: Design by Tulving and Kroll (1995): pre-familiarised and novel items are presented intermixed at critical study for which recognition memory was later tested. Panel B: Design of von Restorff/distinctiveness paradigm: items of the same type/class are presented in lists together with a conceptually or perceptually deviant item (e.g. different font type/colour), memory for which can compared to an item same position in a control list without deviants. Panel C: Rule based design by Greve et al. (2017): At study new scenes were paired with new words, which had the same valence as expected from a previous familiarisation phase (low PE) or the opposite valence (high PE). A forced-choice memory test matched target and foils to be of same valence and equally familiar. Panel D: Reward PE design by De Loof et al. (2018): one, two or four Swahili words are presented as options from which the rewarded word is selected, which manipulated the size of RPE. Panel E: Design by Reggev et al. (2018): Judging whether a noun-adjective is congruent and subsequently testing memory for the nouns. Panel F: Item novelty assessed by presenting objects vs. non-objects or words vs. non-words in Kroll and Potter (1984). Panel G: Mismatch design from Kumaran and Maguire (2006): sequences of objects were represented twice, wherein the second presentation, the order of objects was either unchanged (S_rep_), changed after the first half (S_half_) or completely new (S_new_).

We start with what has been called the classic ‘novelty effect’ in episodic memory (Tulving & Kroll, 1995; though first mentioned by Kinsbourne & George, 1974). In this paradigm, the participant is familiarised with a list of random words. In a second “critical” phase, they see a list of new words, intermixed with some of the familiarised words. Finally, they are presented with a third list of words, and asked to recognise any that came from the critical phase (Fig. 2A). The common finding is that the new words are better recognised than the familiar words, despite the fact that they were presented fewer times in total (note, it is critical that participants are asked to recognise items specifically from the critical phase; if participants are instructed to recognise items studied in either list, memory is better for items that were repeatedly presented (Kafkas and Montaldi, 2015a, Kim et al., 2012).

However, the cause of this ‘classic’ novelty effect has been challenged by alternative explanations, other than a special role for novelty in encoding, such as distinctiveness or source confusion at retrieval (Åberg and Nilsson, 2001, Dobbins et al., 1998, Greene, 1999, Poppenk et al., 2010). In general, these authors attribute the difference in recognition to an impairment for familiar stimuli owing to contextual interference, e.g., “Did I see this stimulus in the familiarisation phase (first list) or study phase (second list)?”, rather than a benefit for the non-familiarised (novel) stimuli, which produces increased false alarm rates and hence overall decreased old/new discrimination. However, other studies controlled for confounds like interference, and reinforced the conclusion of Tulving and Kroll (1995) conclusion. Åberg and Nilsson (2003), for instance, reported a novelty effect for high confidence responses that showed both increases in hit rates and decreases in false alarm rates for novel items, which suggests the effect cannot be explained fully by reduced retrieval accuracy for familiar items, but rather enhanced encoding of novel items. Similarly, Kormi-Nouri et al. (2005) presented distinct encoding tasks to minimize source confusion at retrieval, and still observed better memory for novel over familiar words, consistent with the novelty-encoding hypothesis.

How is this novelty-encoding effect explained by PIMMS? One possibility relates to predictions made by the temporal context. When you encounter the list of random words in the familiarisation phase, you are unable to predict the next word. However, in the critical phase, you notice that some of the words are repeated from the familiarisation phase, so you might start to expect further repetitions. Indeed, if novel items become less expected, they will elicit a greater PE than the familiar words, and therefore become more strongly associated with the context of the critical study phase (hence an example of context surprise). Therefore, when finally asked to recognise words specifically from that phase, you are better able to do so. If this interpretation is correct, then the advantage of these “novel” words should depend on the strength of expectation for repeated words, which could be tested by manipulating the ratio of familiarised to new words in the critical phase. This prediction is consistent with Kafkas and Montaldi (2015a), who showed that the novelty effect reverses when the previously presented words are rare, i.e., it is the unexpectedness rather than relative familiarity that determines memory.

### 3.2 Expectations arising from shared semantics

Another long-standing line of research also uses repetition to build up expectations within the course of the experiment. However, rather than using temporal context to drive the expectation of pre-familiarised items, as in Tulving and Kroll (1995)’s classic novelty effect above, these studies repeat the same class or type of items, thereby generating expectations about the category of the next item (even if not the specific exemplar). Early studies used lists of simple stimuli such as letters, numbers or words, and found memory was superior for deviant (or “oddball”) items than for other items (at the same position in other lists) that were congruent with the context generated by the list (Fig. 2B). This finding is also known as ‘von Restorff effect’, since in one of her seminal studies, von Restorff (1933) presented four pairs of syllables along with one pair of symbols, numbers, letters or patches of colour. The pairs that only occurred once were better recalled immediately afterwards. Although better recall performance is generally thought to be supported by recollection, consistent with PIMMS in terms of contextual predictions at the episodic level, other studies that used recognition performance instead reported stronger familiarity as well (e.g. Kishiyama and Yonelinas, 2003, Rangel-Gomez and Meeter, 2013, von Restorff, 1933). According to PIMMS, increased familiarity would only be expected if the deviant items were also surprising in their perceptual form (see Item Surprise below).

### 3.3 Expectations arising from trained associations or rules

Other studies have varied the strength of predictions by training associations or relations between items. For example, Greve et al. (2017) repeatedly paired a scene with a category of words (Experiment 1) or a specific face (Experiment 2) during a training phase, to establish predictions for what was associated with each scene. In a critical encoding phase, the scene was paired once with a new word/face, and then memory for that new associate was tested in a final cued recall phase (where the cue was the scene and three response options were given, all from the encoding phase, but only one of which was paired with the cue; see Fig. 2C). Memory for the new association was better when the prediction was violated (Experiment 1) or when the prediction that was violated was more precise (Experiment 2), directly supporting a prediction error account.

In a later study, Greve et al. (2019) trained participants with simple rules that related two sets of objects, and found that events (i.e., individual trials with a specific number of each type of object) which violated expectations were better recognised than events that had weaker expectations. This converges with finding from Kafkas and Montaldi (2018a), who asked participants to associate symbols with categories of objects (living/non-living) and reported increased levels of recollection for objects that were unexpected. In another study, using the mismatch paradigm (see section Neuroimaging studies of novelty and surprise in humans; Fig. 2G), it was reported that memory was better for objects when their occurrence violated the sequence shown immediately before (Chen et al., 2015).

While the above studies are consistent with a general pattern of PE driving declarative memory, they also illustrate a limitation of PIMMS, in that it remains unclear what is generating these predictions, since the predictions from trained rules or item-item associations do not obviously come from the spatiotemporal context, nor from (pre-existing) semantic knowledge. The fact that the greater PEs lead to better recollection (e.g., Kafkas & Montaldi, 2018a) suggests they involve the episodic system, but another possibility is that a large PE, regardless of where the prediction comes from, always results in better episodic encoding of the event associated with that error (see below). More generally, it seems likely that multiple predictions, e.g., about which objects will be present in an environment, can arise simultaneously from different sources: long-established semantic knowledge about certain environments (e.g., restaurants), recent rules or patterns observed (e.g., in “situation models” or working memory, like in the Greve et al. (2019), study) or even transient or established associations between objects that tend to co-occur. Distinguishing the roles of these different types of prediction, rather than attributing all to a hippocampal representation of “context”, may be an important future research direction, including the potential for different brain regions being involved.

### 3.4 Expectations arising in reinforcement learning

Another method is to establish predictions for a reward following a stimulus. In the human literature on such “reward PE” (RPE), a stimulus (or class of stimuli) is typically repeatedly paired with a reward (e.g., money), so that future presentations of that stimulus lead to the anticipation of reward, and a RPE is triggered if the reward is not delivered (Fig. 2D). Interestingly, thus far we have been talking about *unsigned* PE (in the context of PIMMS), but with RPE, because the reward has a direction (more or less), one can additionally distinguish *signed* RPE, i.e., positive when the actual reward is bigger than the predicted award, and negative when the actual reward is less. Although the literature on RPE has typically focused on non-declarative learning (e.g. O’Doherty et al., 2004), RPE can also affect declarative memory. De Loof et al. (2018), for example, asked participants to learn Dutch-Swahili word pairs, in which they varied the number of Swahili alternatives for each Dutch word (affecting the precision of the prediction) and the monetary reward (to produce either positive or negative RPE). The data revealed a positive, linear relationship between signed RPE (from negative to positive) and memory performance (see also Calderon et al., 2020). Jang et al. (2019) reported a similar result, though other studies have argued that memory performance is better predicted by unsigned RPE, i.e., its magnitude regardless of sign (Rouhani et al., 2018, Rouhani and Niv, 2019). The relationship between the sign of a RPE and subsequent memory therefore requires further investigation, which might include for example studies in which the reinforcement is aversive rather than rewarding (e.g., when a cue predicts a painful shock rather than money). It is also important to keep in mind the type of memory being tested: whether it is an association between a stimulus and a reward (or an association between two items), which might be declarative and even established on a single trial, but not necessarily episodic in the sense of being bound to a specific spatiotemporal context (e.g., trial in the experiment). Alternatively, it is possible, as suggested above, that a large PE triggers episodic encoding, in addition to strengthening associations between items and/or reinforcers.

### 3.5 Expectations arising from real-world knowledge

In the studies discussed so far, predictions were constructed ‘online’ within the course of the experiment and the results generally endorse the view that contextual surprise enhances memory. In real life, however, context surprise is more likely to arise from expectations that were established over an extended period of time, and some studies draw on this real world knowledge as a source of predictions.

For example, Pine et al. (2018) had participants learn factual information (e.g. “How many weeks did the Falklands war last?”) and found better memory for trials that violated participants’ prior expectations. Similarly, Foster and Keane (2019) presented stories that contained either well-known surprises (e.g. realising that a wallet is missing when walking down the street), less well-known surprises (e.g. realising that a belt is missing in the same scenario) or no surprises at all. The harder it was to intuitively explain a surprise, the better the story was subsequently remembered, which fits well with a PE account.^3^ Similarly, Prull (2015) presented participants with everyday objects in typical or atypical locations, which were either consistent or inconsistent with their schema, i.e., prior knowledge about the structure of the world. The authors showed that recognition accuracy was better for schema-inconsistent trials, and this advantage was driven by recollection. Interestingly, recent studies have started to explore more naturalistic stimuli, such as video clips, whose continuous nature is an even better match of real life experience. In a study by Straube et al. (2014), for example, participants viewed video clips of an actor verbalising a sentence and concurrently performing a congruent gesture, an unrelated gesture or no gesture at all. Sentences with surprising (unrelated) gestures were better remembered than sentences with no gestures. More recent studies have used immersive virtual reality to demonstrate better memory for objects that occur in unexpected locations based on pre-experimental knowledge for where those objects should occur, e.g., within a virtual kitchen (Quent & Henson, 2018).

## 4 Item surprise

An example of item surprise would be encountering a pink sheep, i.e., a familiar object that has most of its expected features except for a few unexpected ones (Fig. 1B). According to PIMMS, this PE between the semantic and feature levels should improve encoding of this new object (in contrast to an unfamiliar animal, which happened to be pink). However, this is at odds with some studies in the literature that report better memory for item-feature binding that is congruent with pre-experimental knowledge. Staresina and Davachi (2006), for example, presented colour–nouns associations and when participants were instructed to create a mental image that combines both (e.g. yellow banana, pink elephant etc.), they were able to recall more plausible than implausible combinations at test. In a similar vein, Reggev et al. (2018) found superior memory for nouns that were previously paired with adjectives consistent with prior expectations (e.g. yellow banana), compared to those in pairs that violated such expectations (e.g. purple banana; Fig. 2E). Some of these cases where memory is better for items that are congruent with prior knowledge (rather than incongruent) might be explained by prior knowledge improving recall at test, e.g., to generate a typical banana from semantic memory, think of its colour as yellow, and then recognise that a yellow banana was presented (a so-called generate-and-recognise strategy; Watkins & Gardiner, 1979). This potential confound can be addressed by presenting response options that are equally congruent or equally incongruent with prior knowledge, e.g. two-alternative forced choice for a yellow banana vs. a green banana, or a purple banana vs. a red banana (see e.g. Quent & Henson, 2018).

These situations demonstrate that novelty/surprise is not the only factor that drives declarative memory encoding, and that sometimes, congruency with prior knowledge (or a “schema”) can boost memory encoding too, even though this is a situation with low PE (as in Fig. 1E). This cannot be explained by PIMMS, and has led some authors to propose that a separate brain system (involving the medial prefrontal cortex) supports the rapid learning of new information that is congruent with prior knowledge (van Kesteren et al., 2012). The existence of two types of learning (one based on large PE and one based on small PE) predicts that memory should be a U-shaped function of expectancy (or schema-congruency), where the highly expected and highly unexpected extremes lead to better memory than the central point of minimal (un)expectedness (a prediction recently confirmed by Greve et al. (2019)). The important implication of this U-shaped function for present concerns is that, when just two conditions are compared (as in many of the studies above), whether memory is better for expected or unexpected information will depend on where those conditions fall on the U-shaped function: if they fall more towards the unexpected extreme, then the condition with greatest unexpectedness will produce better memory, whereas if they fall more towards the expected extreme, then the condition with greatest expectedness will produce better memory. Thus, designs with at least three levels of expectancy measures (i.e. unexpected, neutral and expected) are needed, to appropriately sample both extremes of the U-shape function.

## 5 Context novelty

Context novelty, rather than context surprise, refers to a novel context that makes no clear predictions. This describes the situation in many laboratory studies of memory for random lists of items, where there are minimal predictions about each item before it appears. Though PE is not as high as in the cases of surprise above, where there are (incorrect) predictions, PIMMS still predicts higher PE for familiar than novel items (since former have a more precise likelihood; Fig. 1C).

A different paradigm, but one whose results can be explained in a similar manner, is that used by Kaula and Henson (2020), where some items (faces) were pre-familiarised (primed), and then intermixed with new faces in a second critical phase, before memory for primed and new faces was compared in a final recognition phase (see also Experiment 3 of Greve et al., 2017). While this paradigm is similar to the Tulving and Kroll (1995) paradigm shown in Fig. 2A, an important difference is that Kaula and Henson (2020) paired each face with a unique context (scene) in the critical study phase, rather than the common “temporal” context assumed to apply in Tulving & Kroll’s paradigm. Since the scene-face pairing was arbitrary, the scene imposed no prior on the specific face, whereas it can be argued (as in Context Surprise section above), that the temporal context in the Tulving & Kroll paradigm did impose a prior towards expecting familiar items (depending on the ratio of familiar to new items). This may explain the opposite findings: whereas Tulving and Kroll found better memory for the new items (because they violated the expectation for familiar items), Kaula & Henson found better memory for the familiar items, in the form of better associative memory for the face-scene pairing. The latter is consistent with a greater PE for the primed than new items, owing to a more precise likelihood (in the presence of a flat prior; Fig. 1C). However, further work is needed to distinguish this PIMMS account from an attentional resource account, where priming results in less attention being devoted to processing the item, and more attention being devoted instead to processing its context (background scene; see Kaula & Henson, 2020, for further discussion).

## 6 Item novelty and complete novelty

The converse situation to context novelty is item novelty, where a completely new item is encountered that has no pre-existing semantic representation, i.e. a flat likelihood. If the context also makes no predictions, i.e., the prior is also flat (i.e., the case of “complete novelty” in Fig. 1F), then there should be minimal encoding. This is consistent with a number of empirical studies that use random lists of items (i.e., contexts with no strong priors) and compare memory for known vs. unknown items, such as familiar vs. unfamiliar symbols (Cycowicz, 2019, Cycowicz and Friedman, 2007), non-words vs. words (Belleville et al., 2011, Gardiner and Java, 1990, Perfect and Dasgupta, 1997) or unknown vs. known melodies (Belleville et al., 2011, Java et al., 1995). As expected from PIMMS, episodic memory for the unknown items is worse.^4^ In the situation where the likelihood is flat but there is a precise prior, i.e., when unknown items are presented in a context that predicts certain known items, PIMMS predicts there should be some encoding driven by the PE (Fig. 1D). We are not aware, though, of any experiments that have compared this situation to the complete novelty situation of a flat prior.

Returning to the situation of complete novelty (Fig. 1F), this is interesting because PIMMS predicts little encoding, despite maximal novelty. According to PIMMS, the sensory systems (e.g. occipito-temporal cortex) are able to represent the perceptual details of completely novel items (to the extent that the incoming sensations can be processed at all), but the lack of prior knowledge in the semantic or episodic level prevents any effective predictions. While the amount of encoding in such situations may be minimal, it is clear that we must be able to learn something, in order to “bootstrap” learning of new items in new contexts. However, it may be that many such learning episodes are needed before predictions can be established, so that memory for any single episode is poor. Furthermore, other factors may also come into play in these situations of maximal novelty, such as increased attention/arousal, which may affect encoding of other information nearby in time, as we discuss next.

## 7 Effects of novelty on memory for surrounding information

So far, we have talked about memory for the novel information itself. In other situations, novelty/surprise may impact memory for additional types of incidental information that surround novel information (in time and/or space), even if that other information is relatively unrelated and not necessarily novel itself. Let us return to our example in which you encounter a flock of sheep on your way to work, which was highly surprising. There is good evidence that you will remember this event better than other morning commutes. However, what is it that will be stuck in your memory? Is it just the unusual event of seeing sheep, or will you show a benefit for other contextual information that was present at the same time, such as what song was playing in the radio, what clothes you were wearing or whether the sun was shining? In addition, are you also more likely to remember other moments that happened right before or after the encounter of the sheep, such as whether you did the school run that morning or with whom you had a meeting once you arrived at work?

### 7.1 Effects at the level of source information

One related question for PIMMS is whether PE only enhances learning of associations between the predictor (e.g., item) and the predicted (e.g. features) – i.e. learning that is “local” to the cause of the PE – or does it trigger a “global” increase in learning which also enhances memory for other incidental information that happens to co-occur with a PE? An example of memories that contain rich incidental, episodic information are “flashbulb” memories, which lead to clear recollection of the context surrounding a particular event, such as where one first heard the news of the 9/11 terrorist attacks (Brown & Kulik, 1977; Hirst et al., 2015). Flashbulb memories may be driven in part by the extreme novelty or surprise of the event – which would suggest a global effect of PE, rather than one specific to the cause of the PE - though it is clear that emotional factors and the significance of the event also play an important role. Indeed, there is evidence that surprise per se is not necessary for flashbulb memory (Coluccia et al., 2010).

In mundane laboratory studies, this incidental contextual information is often called “source information”, which can range from where on the computer screen an item was presented, to the mental thoughts that occurred at the same time (Johnson et al., 1993). For example, Kim et al. (2012) found that memory for source information (e.g., the location of an item or the background colour) was better for novel compared to repeated items (where repeated items were presented before the colour/location association in a pre-exposure phase)^5^. Likewise, Greve et al. (2019) found that memory for incidental information (in the sense that it was not task-relevant) was better when events violated the learned rule. In line with these findings, other studies report that low-frequency (and hence more surprising) words are associated with better source memory (i.e., word was spoken in a male or female voice) than high-frequency words (Guttentag & Carroll, 1994), or the mental operations performed (Guttentag and Carroll, 1997, Ye et al., 2019). On the other hand, some studies (e.g. Uncapher et al., 2011) report the opposite pattern of results, with worse source memory (for presentation side on the screen) for invalidly-cued than validly-cued trials in a Posner cuing task. It remains to be seen whether other factors beyond novelty, e.g. changes in attention, could explain these contradicting findings.

### 7.2 Temporal extent of the effect

The previous section considered the effect of novelty on simultaneous source information. However, what about information experienced shortly before or after a novel event? There is evidence that novelty can exert a beneficial effect both retroactively and proactively, so as to “spill-over” to stimuli that are not novel themselves, but are simply encountered in close temporal proximity. This effect, inspired by the synaptic tag and capture theory (Frey & Morris, 1997) was first studied extensively in animals (e.g., Ballarini et al., 2009, Moncada and Viola, 2007, Wang et al., 2010). For example, it is well documented that when a rodent is placed in a novel environment, memory for information learned immediately before or after that event is boosted (Wang et al., 2010). Similar effects have since been reported in humans (Ballarini et al., 2013, Bunzeck and Düzel, 2006, Fenker et al., 2008, Ramirez Butavand et al., 2020, Schomaker, Roos, et al., 2014; but see Biel & Bunzeck, 2019). Fenker et al. (2008), for example, reported that participants who viewed novel versus familiar scenes showed better memory for unrelated words that were presented either before or after a scene (though see Biel & Bunzeck, 2019, for a failure to find this effect). A proactive memory benefit has also been reported by Schomaker, van Bronkhorst, et al. (2014) who employed immersive virtual reality and demonstrated enhanced memory for words that were learned immediately after exposure to a novel environment (analogous to the experiments on rodents). Similarly, Wittmann et al. (2007) reported superior memory for items that were preceded by a cue that predicted novelty, irrespective of whether the item itself was novel. Most impressively, the novelty effect on surrounding information has been tested in real-world educational setting by Ballarini et al. (2013), who demonstrated that teaching novel content to primary school children in an engaging science or music lesson, can boost memory for unrelated verbal and pictorial material studied up to one hour (but not four hours) before or after the lesson. This has been replicated in another set of high school students (Ramirez Butavand et al., 2020). These retroactive and proactive effects suggest that novelty causes short-term changes in general synaptic plasticity (see below), which facilitate consolidation processes (at least in the case of retroactive effects), in addition to the encoding processes we have considered so far. Moreover, the findings from Schomaker, Roos, et al., 2014, Schomaker, van Bronkhorst, et al., 2014, Fenker et al., 2008 were evident in recall and recollection which is in line with the idea that novelty enhances memory by engaging hippocampal encoding/consolidation processes (Kafkas & Montaldi, 2018b).

## 8 Novelty and event boundaries

In addition to how well we remember information, novelty and surprise also appear to play an important role in parsing or segmenting our continuous experience. In contrast to most laboratory experiments that show discrete events/items/trials, our experiences in the real world are derived from a continuous stream of sensory input, during which our brains are constantly trying to predict what will be perceived next. According to “event segmentation theory” (Reynolds et al., 2007, Zacks et al., 2007), sudden changes in the input stream can evoke a violation of such predictions, which is believed to create boundaries that mark the beginning and the end of an event. In other words, prediction errors also act as a gating mechanism that parses our continuous perception into discrete memories.

This notion is supported by Rouhani et al. (2019), who associated scenes with a monetary reward and found that memory was worse for associations that spanned across high reward prediction error (RPE) trials than low RPE trials. This is in line with a range of studies that confirm memory is better for items that belong to the same event (DuBrow and Davachi, 2013, DuBrow and Davachi, 2016, Ezzyat and Davachi, 2014, Heusser et al., 2018, Horner et al., 2016). Memory for event boundaries themselves has been reported to be superior (Rouhani et al., 2019, Swallow et al., 2009) and objects encountered at boundaries show stronger item-context binding (for images on colour backgrounds) compared to non-boundary objects (Heusser et al., 2018). This strengthens the view that an abrupt context shift in our environment can elicit a PE, which leads to boundaries that influence the formation, strength and structure of how we remember events. Indeed, neuroimaging has revealed peaks in activity in the hippocampus at event boundaries (Ben-Yakov & Henson, 2018), and the level of this “event offset” activity seems to correlate with how well the preceding event is remembered (Ben-Yakov & Dudai, 2011). Animal research suggests that such “spill-over” effects last a fixed period of time (see Okuda et al., 2020), so an interesting question for human studies is whether “spill-over” effects extend only as far as the surrounding event boundaries (Ben-Yakov et al., 2020).

## 9 Brain systems of novelty and surprise

So far, we have talked about the behavioural predictions of PIMMS. There is also a wealth of neuroimaging studies on humans (e.g., using PET, fMRI, EEG) that address the brain regions involved and/or the temporal dynamics of novelty effects, and an even larger literature on animal studies of the neurobiology of novelty and memory. We only scratch the surface of these literatures here, in an attempt to bring out the clearest findings in relation to some of the distinctions outlined in the previous sections.

### 9.1 Neuroimaging studies of novelty and surprise in humans

Context surprise has consistently been associated with the medial temporal lobe (MTL), and the hippocampus in particular, across a wide range of neuroimaging studies (e.g. Axmacher et al., 2010, Chen et al., 2015, Daselaar et al., 2006, Dolan and Fletcher, 1997, Grunwald et al., 1998, Grunwald and Lehnertz, 2003, Guitart-Masip et al., 2010, Knight, 1996, Köhler et al., 2005, Maass et al., 2014, Poppenk et al., 2008, Rutishauser et al., 2006, Tulving et al., 1996). Stronger hippocampal activity for context surprise was first reported in an early PET study by Tulving et al. (1996) who presented novel items intermixed with familiar items (see Fig. 2A). Concerns that this truly reflects activation for novel events rather than habituation to familiar events were overcome in later studies that tested “match-mismatch” paradigms (Kumaran and Maguire, 2006, Kumaran and Maguire, 2007, Kumaran and Maguire, 2009). These paradigms contrasted repeated sequences of objects (e.g., ABCD) with partially rearranged sequenced in which only the first two objects remaining intact (e.g., ABDC) and fully scrambled sequences (e.g., DACB; see Fig. 2G). Compared to repeated sequences (i.e., maximal habituation), or fully scrambled sequences (i.e., maximal novelty), hippocampal activity was greater for partially rearranged sequences, for which predictions generated by the first two objects (AB) were violated by object D which occurred instead of C (i.e., maximal PE). This reaffirmed that the hippocampus tracks context surprise/PE, rather than indexing novel arrangements per se. Various other studies, assessing real-world predictions (Kang et al., 2009, Pine et al., 2018, Schiffer et al., 2012, Straube et al., 2014) and RPE (Davidow et al., 2016; though see Wimmer et al., 2014) corroborated the conclusion that the hippocampus processes contextual surprise. Importantly, not all of these studies related the novelty effects to subsequent memory, though those that did (e.g. Chen et al., 2015, Kirchhoff et al., 2000) generally find the same regions that show novelty/surprise, also predict subsequent memory.

These findings are in line with the PIMMS framework, which predicts strong hippocampal activity for context surprise (Fig. 1A), and that this should predict subsequent episodic memory. It should be noted, though, that empirical studies have also linked additional brain regions to contextual surprise, most prominently the locus coeruleus (LC), the substantia nigra, striatum and ventral tegmental area (Clos et al., 2019, Fenker et al., 2008, Guitart-Masip et al., 2010, Hollerman and Schultz, 1998, Kamiński et al., 2018, Mikell et al., 2014, Murty et al., 2016, Schott et al., 2004, Wittmann et al., 2007).

Studies that use event-related potentials (ERPs) to examine context surprise, report deflections of a central-parietal ERP component known as the P3, elicited 300–400 ms after stimulus onset (e.g. Friedman et al., 2001, Polich, 2007). Stronger P3 modulation has been reported for deviant relative to standard items in oddball paradigms (Nieuwenhuis et al., 2011, Polich, 2007). Notably, the P3 amplitude is sensitive to the difference in frequency of occurrence and level of deviation between standard and deviant items (Ranganath and Rainer, 2003, Nieuwenhuis et al., 2011), which endorses the view that this component reflects expectancy but not novelty per se. However, it is also important to relate these ERP novelty effects to memory encoding and here the current literature does not offer a coherent picture. While some studies show the P3 amplitude is linked to subsequent recall (Butterfield and Mangels, 2003, Fabiani et al., 1990, Fabiani and Donchin, 1995, Kamp et al., 2013; but see Rangel-Gomez & Meeter, 2013), others failed to observe P3 subsequent memory effects in recognition paradigms (Fabiani & Donchin, 1995). This discrepancy might be explained by the different types of memory tests, which are subserved by different processes. However, hippocampal intracranial EEG shows a component similar to the P3 that is related to subsequent memory effects (Axmacher et al., 2010), lending support for a general relationship between P3, hippocampus and memory function (for a review see Fonken et al., 2020).

In addition to context surprise, PIMMS predicts hippocampal activity for context novelty or item novelty, albeit lower, since it is driven by a smaller PE than for context surprise (Fig. 1). This has received some empirical support from intracranial ERP studies that show evoked potentials from hippocampus (N300/N350 and P600-like responses) are less pronounced for nonsense objects (item novelty) than real objects (Vannucci et al., 2003, Vannucci et al., 2008) in a context that holds no strong predictions. Interestingly, a difference between real objects and nonsense objects was only observed for patients with intact but not with impaired visual memory (Vannucci et al., 2008). However, some studies report the opposite finding, with stronger hippocampal activity for non-words than words, at least when tested with a lexical decision task (Graves et al., 2017, Mattheiss et al., 2018; though not when tested in a passive viewing condition, Braun et al., 2015). Further studies are needed to delineate whether task difficulty might confound the findings of item novelty in a lexical decision task because non-words might require more processing time than words (Graves et al., 2017).

Other ERP studies have examined item novelty. For example, a study by Cycowicz and Friedman (2007) reported a smaller P3 for repeated familiar symbols compared to repeated novel symbols, which was in turn associated with worse memory for the novel symbols (also see Cycowicz, 2019). This is also the case in tests using illegal non-words, distorted pictures or unknown sounds (for reviews see Friedman et al., 2001, Rugg and Doyle, 1994). More importantly, the subsequent memory effect was larger for familiar than unfamiliar symbols (Cycowicz, 2019). These studies confirm that item novelty is associated with less effective encoding compared to familiar items. More generally, these ERP signatures suggest that it might be important to differentiate context surprise from item surprise, and possibly context/item surprise from context/item novelty (also see Schomaker, Roos, et al., 2014, Schomaker and Meeter, 2015).

### 9.2 Neurobiological models of novelty and surprise

So far, we sought to explain how novelty and surprise determine the fate of new declarative memories through the lens of PIMMS. Although PIMMS offers a framework for thinking about novelty and surprise, as well as making predictions for behaviour, it remains largely silent as to what brain mechanisms, neurotransmitters and molecular processes might underpin such behaviour. These mechanisms have been addressed by several neurobiological models of novelty and surprise in the animal literature. However, it is often difficult to reconcile those models with the data reviewed above, because of disparities in the terminology and nature of the paradigms used in the animal and human literature (see also Schomaker, 2019).

In line with human neuroimaging studies, most models propose that the hippocampus plays a central role, but make different claims about the neural circuits involved in processing novelty and surprise. Schomaker and Meeter (2015), for example, suggest stimulus and contextual novelty (or what we call item novelty and context novelty) engage the amygdala and enhance perceptual sensitivity, through the substantia nigra/ventral tegmental area (SN/VTA) and modulation of long term potentiation (LTP). More importantly, surprise, rather than novelty, is thought to engage a separate system including the anterior cingulate cortex (ACC), which activates the LC and norepinephrinergic system.

On the other hand, Duszkiewicz et al. (2019) propose that the hippocampus receives dopaminergic modulation from both the VTA and the locus coeruleus (LC). Indeed, the direct connection between hippocampus and LC might be even more critical than the indirect link to the VTA (Takeuchi et al., 2016). Duszkiewicz et al. (2019) distinguish “common novelty”, i.e. experiences that share some aspects with past experiences from “distinct novelty”, i.e. an experience that shares only few aspects with past experience (such as seeing the ocean for the first time). The latter is probably related closest to our term ‘complete novelty’, which is claimed to activate the LC and initiate consolidation processes in the hippocampus, which creates a memory trace associated with better recollection. Nevertheless, these data do not align well with PIMMS and the human literature, which shows rather poor encoding for item/complete novelty (see earlier), which might reflect inconsistencies in the translation of animal and human studies.

Interestingly, an influential framework (Düzel et al., 2010, Lisman et al., 2011, Lisman and Grace, 2005) suggests the hippocampus detects surprise (though referred to by the authors as “novelty”) of any type, and sends a signal to the SN/VTA via the striatum, which in turn releases dopamine in the hippocampus. Within the SN/VTA, these signals modulate phasic and tonic activity patterns. Düzel et al. (2010) speculated that phasic dopamine induces the production of plasticity-related products (PRPs, see below), while tonic dopamine activity increases the probability of phasic bursts, which lower threshold for learning. An important consequence of experiencing surprise according to this model is that it increases motivation and drives exploration (Düzel et al., 2010). Such effects are largely neglected in the human literature, but might play an important role and enhance memory encoding/consolidation besides PE.

Kafkas and Montaldi (2018b) propose that the anterior hippocampus is crucial for detecting surprise (referred to by the authors as “novelty”), at which point it sends a signal to other modulatory regions (e.g. midbrain and striatum). This includes release of acetylcholine, which induces pupil constriction. Interestingly, Naber et al. (2013) also link pupil constriction during encoding of surprising items to better subsequent retrieval. This effect occurs regardless of whether an item is a target or non-target in the encoding task, although task relevance of targets further modulates pupil size (Kafkas and Montaldi, 2015a, Kafkas and Montaldi, 2015b, Otero et al., 2011). Importantly, other studies show the opposite finding, i.e., pupil dilation for surprise (Kafkas and Montaldi, 2015a, Kloosterman et al., 2015, Preuschoff et al., 2011) and curiosity (Kang et al., 2009). Pupil dilation is associated with the sympathetic nervous system, i.e., acetylcholine and noradrenaline, while pupil constriction is regulated by the parasympathetic nervous system, which engages acetylcholine only. These data seem to indicate that two distinct brain regions and neurotransmitter systems could be engaged by surprise, hence further differentiation of context surprise might be needed to distinguishes surprise driven either by a rare occurrence of an item (i.e. Kafkas & Montaldi, 2015a) from surprise that is generated by expectations from previous presentations (i.e., Naber et al., 2013). More work is needed to distinguish these possibilities.

Many of the models mentioned so far base their assumptions on key findings and ideas in the animal literature, such as the “behavioural tagging theory” (BTT; Ballarini et al., 2009, Moncada and Viola, 2007). BTT itself is derived from the synaptic “tag-and-capture” theory (Frey and Morris, 1997, Redondo and Morris, 2011). This theory posits that the mechanism underlying the maintenance of late-stage LTP involves two processes. In the first step, a synapse is tagged as the result of recent input. After tagging, the synapse then captures so-called plasticity-related products (PRPs). This leads to the induction of lasting structural change in the synapse. A seminal experiment by Frey and Morris (1997) showed, when a synapse receives weak tetanisation, the synapse is tagged and early-LTP induced. However, without captured PRPs, early-LTP does not automatically become late-LTP, but strong tetanisation of a different synapse on the same neuronal population can do just that by providing PRPs to the weakly stimulated synapse. In this way, according to BTT, experiencing surprise (as novelty is operationalised in these studies) can have a similar effect as strong tetanisation and induce late-LTP after only weak stimulation (Li et al., 2003, Straube et al., 2003, Straube et al., 2003).

Interestingly, a tag is believed to last approximately 90 min (Redondo & Morris, 2011), which means there is a critical time window during which a strong tetanisation (e.g., from novelty) has to be experienced to cause long-lasting memory change (Ballarini et al., 2009, Moncada and Viola, 2007). The time window is further influenced by other task characteristics and might follow a nonlinear function (Moncada et al., 2015). More importantly, this mechanism of time limited PRPs might explain the novelty/surprise effects for temporally-surrounding information discussed earlier in the human literature (see section Temporal extent of the effect). The human studies, however, show that the surprise-induced memory advantage for surrounding information can sometimes occur within a few seconds or minutes of the learning experience (e.g. Bunzeck and Düzel, 2006, Schomaker, Roos, et al., 2014), which is too fast for the consolidation mechanisms involving PRPs to unfold. Future studies are needed to pinpoint the precise neurobiological mechanism underlying these effects in humans.

Finally, the interplay between PE, protein synthesis and consolidation is also captured in the phenomenon of reconsolidation, by which reactivated memory traces are destabilized and altered (Hardt, Einarsson, & Nader, 2010). This process depends on behavioural tagging (Rabinovich Orlandi et al., 2020), and is thought to require surprising events, i.e., PE, in order for reconsolidation to be effective. While this has been shown for non-declarative fear conditioning Sevenster et al., 2013, Sinclair and Barense, 2018 propose that it can occur in human declarative memory too. They showed that video clips depicting action-outcomes are associated with higher rates of memory intrusions when the videos were surprisingly cut-off before the action, which they attributed to surprise that allowed memory traces to be modified by reconsolidation.

## 10 How does PIMMS fare?

While PIMMS can explain some of the situations where surprise improves declarative memory, and some situations where simple novelty does not (or even produces worse memory), it clearly has several limitations. Foremost, PIMMS is a framework for thinking about different types of novelty (perhaps at Marr’s “algorithmic” level; Marr, 1971), and while tentative steps have been made to relate some of the layers in the assumed hierarchy to certain brain regions, it is not a theory about the underlying neuronal mechanisms (i.e., at Marr’s “implementation” levels). As reviewed in the above section on neurobiology, there are many complex processes involved in LTP, consolidation and various neurotransmitters that are also potentially affected by novelty, but about which PIMMS is silent. Moreover, though we have currently focused on just three levels in the hierarchy (the episodic, semantic and perceptual), in order to simplify and make contact with most of the human behavioural work on declarative memory, there are likely to be many more levels needed, for example to explain associative novelty, in terms of predictions between two objects that have been paired repeatedly. There are often also “meta-predictions” operating, such as whether or not surprise is itself expected. This has been studied in the context of volatile versus stable environments (Yu & Dayan, 2005), where a surprising event in a volatile (ever-changing) environment is less surprising than one occurring in a stable environment. A further consideration that is not fully addressed here is the role of attention in boosting memory for novel or surprising events. While attention may be a necessary mediator for improving memory, we do not think it is a sufficient explanation, because something else (e.g., PE) first has to cause a change in attention.

More importantly, there are also situations, mentioned above, where the behavioural evidence does not conform to PIMMS’s predictions. These include events that are highly congruent with expectations (or “schemas”; for review see Ghosh & Gilboa, 2014), and so elicit minimal PE and should not be encoded, and yet can still be recalled better (even when controlling for the benefits of schema at retrieval). This led to the proposal of a second brain system specialised for rapid consolidation of schema-congruent events (Alba and Hasher, 1983, Atienza et al., 2011, Cycowicz et al., 2008, Liu et al., 2018). Another situation that is problematic for PIMMS is when unknown objects are encountered in a novel environment that provides few expectations (i.e., flat prior and likelihood), such as a rodent entering a novel arena with novel objects (Tse et al., 2007), where research has demonstrated that this experience boosts memory for surrounding information. Not only does this situation produce negligible PE according to PIMMS, but PE-driven learning in PIMMS is assumed to occur at the time of encoding, so it is silent on processes (like those assumed by BTT) that improve memory for information occurring earlier or later in time, or on memory improvements that only emerge after a period of consolidation. While these cases could be used to indicate that PIMMS is not a good model of how novelty affects memory, we prefer to use these cases to argue that PE-driven memory encoding is not sufficient to explain the effects of all types of novelty, and those situations where PE-driven memory encoding cannot explain the data may be precisely those situations in which the brain needs (i.e., has evolved) other mechanisms by which novelty can influence memory. Clearly, there is an important challenge to develop unified theories that can explain the full and diverse range of ways in which novelty and memory interact.

## Funding statement or declaration of conflicting interests

This work was supported by the United Kingdom Medical Research Council (SUAG/046 G101400). The Gates Cambridge Trust funds JAQ’s PhD studentship. The authors declare no conflicting interests.

## CRediT authorship contribution statement

**Jörn Alexander Quent:** Conceptualization, Visualization, Writing - original draft, Writing - review &amp; editing. **Richard N. Henson:** Conceptualization, Visualization, Writing - original draft, Writing - review &amp; editing. **Andrea Greve:** Conceptualization, Visualization, Writing - original draft, Writing - review &amp; editing.

## Footnotes

1 More precisely, the PE that drives learning is the divergence between the prior and the posterior, on the assumption that the initial PE between the prior and the likelihood is what drives perception (activity changes), which seeks to minimise this PE over a few hundred milliseconds (producing the posterior distribution), leaving the residual PE that drives synaptic change instead (Henson & Gagnepain, 2010). For simplicity though, we assume here that the posterior is close to the likelihood, such that the qualitative implications are the same.

2 Note that that predictions in PIMMS are generally implicit (automatic, unconscious), though they could also be explicit (conscious) and/or generated intentionally. PIMMS does not currently distinguish between these, and makes the same (experimental) prediction of improved memory when PE is high, regardless of whether the (theoretical) prediction is implicit or explicit.

3 Interestingly, this advantage for context surprise appears to be moderated by the degree of individual curiosity, according to Kang et al. (2009), who asked participants to firstly guess the correct answer to a question and secondly rate how curious they were to learn the true outcome. Recall accuracy for incorrectly guessed responses, i.e. novel or surprising facts, increased linearly with curiosity (also see Gruber & Ranganath, 2019).

4 Note that the flip case of this situation is that the memory for familiar items is better because of context surprise, as described in previous section, owing to the PE arising where the prior is flat but the likelihood is precise (Fig. 1C).

5 Note this finding appears to contradict that of the Kaula and Henson (2020) study described earlier; for possible reasons, see Discussion section of Kaula and Henson (2020).
